# Production and secretion of functional SARS-CoV-2 spike protein in *Chlamydomonas reinhardtii*

**DOI:** 10.3389/fpls.2022.988870

**Published:** 2022-09-20

**Authors:** Anna Maria Kiefer, Justus Niemeyer, Anna Probst, Gerhard Erkel, Michael Schroda

**Affiliations:** Molecular Biotechnology & Systems Biology, TU Kaiserslautern, Kaiserslautern, Germany

**Keywords:** synthetic biology, glycoproteins, biopharmaceuticals, microalgal production platform, furin-like protease

## Abstract

The spike protein is the major protein on the surface of coronaviruses. It is therefore the prominent target of neutralizing antibodies and consequently the antigen of all currently admitted vaccines against SARS-CoV-2. Since it is a 1,273-amino acids glycoprotein with 22 N-linked glycans, the production of functional, full-length spike protein was limited to higher eukaryotes. Here we report the production of full-length SARS-CoV-2 spike protein – lacking the C-terminal membrane anchor – as a secreted protein in the prefusion-stabilized conformation in the unicellular green alga *Chlamydomonas reinhardtii*. We show that the spike protein is efficiently cleaved at the furin cleavage site during synthesis in the alga and that cleavage is abolished upon mutation of the multi-basic cleavage site. We could enrich the spike protein from culture medium by ammonium sulfate precipitation and demonstrate its functionality based on its interaction with recombinant ACE2 and ACE2 expressed on human 293T cells. *Chlamydomonas reinhardtii* is a GRAS organism that can be cultivated at low cost in simple media at a large scale, making it an attractive production platform for recombinant spike protein and other biopharmaceuticals in low-income countries.

## Introduction

In late 2019, the severe acute respiratory syndrome coronavirus 2 (SARS-CoV-2) was identified as the causative agent for the coronavirus disease 19 (COVID-19) (Zhu et al., 2020). Since then, the virus is spreading throughout the world, causing not only humanitarian but also severe economic crisis. One of the most important proteins for the pathogenicity of the SARS-CoV-2 virus is the trimeric spike protein on its surface (Yao et al., 2020). This 1,273-amino acids protein contains 22 N-linked glycans and only traces of O-linked glycans (Watanabe et al., 2020). Each spike protein monomer is built of two main subunits S1 and S2, with S1 mediating receptor binding and S2 membrane anchoring (Cai et al., 2020; Walls et al., 2020; Wrapp et al., 2020). Cleavage between the S1 and S2 subunits occurs during spike protein synthesis in the Golgi compartment by a furin-like protease at a multi-basic cleavage site, leaving the S1 and S2 subunits linked by non-covalent interactions (Cai et al., 2020; Hoffmann et al., 2020a). The S1 subunit contains the receptor binding domain (RBD), which binds to the cellular surface receptor angiotensin-converting enzyme 2 (ACE2) (Hoffmann et al., 2020b; Letko et al., 2020). Binding to ACE2 requires at least one RBD in the spike protein trimer to be in the ‘up’ conformation, representing the receptor accessible state, while the ‘down’ conformation is the receptor-inaccessible state. Accordingly, roughly half of the ∼25 spike protein trimers present on the surface of a virion are in the “one RBD up” conformation (Yao et al., 2020). After binding of the RBD to the ACE2 receptor, structural changes occur in the spike protein trimer that expose the S2’ sites located immediately upstream of the fusion peptide, leading to cleavage by the transmembrane protease serine 2 (TMPRSS2) (Matsuyama et al., 2010; Bestle et al., 2020). S2’ sites can also be cleaved by cathepsin L in the endosome after endocytosis of virions (Simmons et al., 2005). Cleavage at S2’ leads to the shedding of S1 subunits and triggers a cascade of folding events resembling a ‘jackknife mechanism’, during which the fusion peptides are inserted into the target cell membrane. Further folding bends the viral and host membranes toward each other, leading to membrane fusion and viral entry (Cai et al., 2020; Jackson et al., 2021).

The spike protein is the major protein on the virion surface and is central for host cell invasion, which is why it is the prominent target of neutralizing antibodies. Therefore, all currently admitted vaccines use the spike protein as antigen (Krammer, 2020; Jackson et al., 2021; Li et al., 2021). While mRNA- and vector-based vaccines have proven efficient and fast to establish, they do have side effects including heart inflammation and blood clots. Moreover, they are difficult to produce and to handle in lower-income countries (Li et al., 2021). In contrast, recombinant protein based vaccines have less side effects and are easier to produce once their production platform is established (Dolgin, 2021). While potent neutralizing antibodies bind to the RBD, the RBD lacks other neutralizing epitopes present on the full-length spike protein (Krammer, 2020; Jackson et al., 2021; Li et al., 2021). Hence, it appears advisable to produce the prefusion-stabilized, full-length spike protein for protein-based vaccines and for serological tests. Accordingly, a phase III clinical trial for the Novavax NVX-CoV2373 vaccine based on recombinant prefusion-stabilized full-length spike protein trimers was recently completed with the vaccine showing 90% protection against COVID-19 (Dunkle et al., 2021). Because of its size and its 22 N-linked glycans, the production of full-length spike protein has been reported in higher eukaryotes including mammalian, insect and plant cells (Zhou et al., 2006; Bangaru et al., 2020; Hoffmann et al., 2020b; Walls et al., 2020; Watanabe et al., 2020; Wrapp et al., 2020; Dunkle et al., 2021; Ward et al., 2021; Pillet et al., 2022), while yeast and microalgal expression hosts have been limited to the production of spike protein fragments, mostly the RBD (Chuck et al., 2009; Arbeitman et al., 2020; Berndt et al., 2021; Dalvie et al., 2021; Malla et al., 2021; Slattery et al., 2022). Moreover, in microalgal expression hosts, the RBD was expressed as a cytosolic or ER-resident protein, requiring purification from whole cells (Berndt et al., 2021; Malla et al., 2021; Slattery et al., 2022). Also, the purification of virus-like particles (VLPs), bearing prefusion-stabilized, full-length spike protein from transiently transfected *Nicotiana benthamiana* leaves, requires tissue homogenization and several chromatographic purification steps (D’Aoust et al., 2010; Ward et al., 2021; Pillet et al., 2022). Importantly, plant-produced VLP vaccine elicited neutralizing antibody production in humans and rhesus macaques and was well tolerated, underscoring the suitability of plant models for vaccine production (Ward et al., 2021; Pillet et al., 2022). The development of simple production platforms for the secretion of complex therapeutic glycoproteins such as the full-length spike protein is desirable to facilitate their production in low-income countries. Microalgae appear to be a good choice here, as they can be grown at large scale in very simple media and do not produce toxic compounds (Rosales-Mendoza et al., 2020). In general, glycosylation is essential for protein folding, stability, and functionality and is the most eminent post-translational modification in biopharmaceuticals (Lingg et al., 2012).

Here we demonstrate the production and secretion of the full-length ectodomain of the SARS-CoV-2 spike protein in the unicellular green alga *Chlamydomonas reinhardtii*. We show that the protein can be enriched by ammonium sulfate precipitation from the culture medium and is functional, as judged by its ability to interact with human ACE2.

## Materials and methods

### Strains and culture conditions

*Chlamydomonas reinhardtii* strain CC-4533 (cw15, mt-), harboring an insertion in the *SRTA* gene (LMJ.RY0402.148523) (Neupert et al., 2020), was obtained from the Chlamydomonas library project (Li et al., 2016). This strain was used as it has been shown to express transgenes as well as the UVM strains (deriving from the cell wall-deficient cw15-302 parent) (Neupert et al., 2020) but contains cilia and more cell wall than the UVM strains, rendering it more robust towards shearing forces occurring in photobioreactors. Cells were grown in 10-mL cultures in Tris-Acetate-Phosphate (TAP) medium (Kropat et al., 2011) on a rotatory shaker at a constant light intensity of ∼40 μmol photons m^–2^ s^–1^. Transformation was performed with the glass beads method as described previously (Niemeyer et al., 2021) with constructs linearized with *Not*I. Transformants were selected on 100 μg/mL spectinomycin (S4014, Sigma-Aldrich). Cell counts were performed with a Z2 Coulter Counter (Beckman).

To validate the functionality of SARS-CoV-2 S-protein, HEK293T cells that stably overexpress hACE2 and hTMPRSS2 (293T + AT; CL0015 VectorBuilder) and non-transfected HEK293T cells (293T; ATCC, CRL-3216™) were used. The cells were maintained in Dulbecco’s Modified Eagle’s Medium (DMEM) supplemented with 10% fetal calf serum and antibiotics (500 μg/mL neomycin (8668.2, Carl Roth), 7.5 μg/mL blasticidin (CP14.2, Carl Roth), 1.5 μg/mL puromycin (A2856, AppliChem) for 293T + AT and 65 μg/mL penicillin G (31749, Serva), 100 μg/mL streptomycin sulfate (0282, VWR) for 293T) in humified atmosphere at 37°C and 5% CO_2_.

### Expression of RBD-sfGFP

As a control for the activity assays, RBD-sfGFP was expressed in 293T cells. The cells were seeded into 175 cm^2^ flasks and incubated until they reached 60% confluence. Transfection was performed using polyethyleneimine (branched, 408727, Sigma-Aldrich). For this, 45 μg of plasmid DNA (pcDNA3-SARS-CoV-2-S-RBD-sfGFP (Chan et al., 2020), http://n2t.net/addgene:141184, kindly provided by Erik Procko) were incubated with 2 mL of DMEM. After 5 min, 805 μL polyethyleneimine (1 mg/mL in H_2_O) was added, immediately mixed and incubated for 10 min. The culture medium was removed from the cells and the mixture was then carefully added. After two hours, the medium was replaced by fresh, serum-free medium without phenol red (P04-710629, PAN Biotech). Expression took place for three days. The culture medium containing RBD-sfGFP was then collected and centrifuged (1,000 g, 10 min, 4°C), and concentrated with centrifugal filters (MWCO 10 kDa; Amicon^®^ Ultra-15).

### Cloning

**FIGURE 1:**
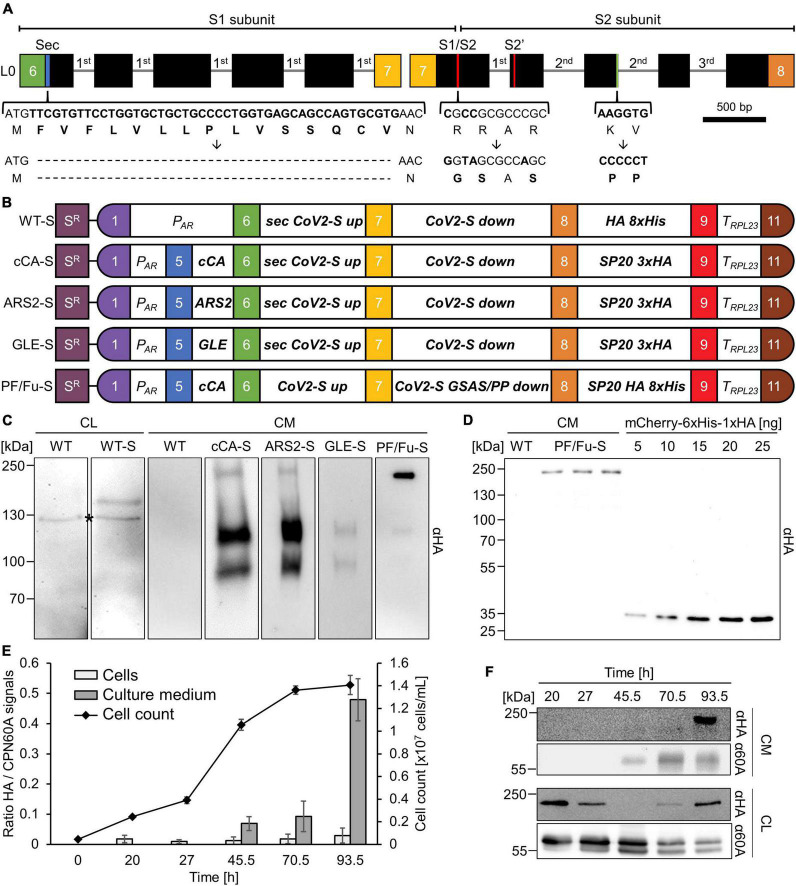
Constructs for the production and secretion of the SARS CoV-2 spike protein in Chlamydomonas. **(A)** Level 0 parts encoding the spike protein. Introduced *RBCS2* introns are shown as gray lines and exons as black boxes. Braces indicate modified regions with resulting amino acid modifications. Colored boxes represent MoClo fusion sites. **(B)** Level 2 devices containing the *aadA* cassette (S*^R^*) and transcription units for spike protein expression. P_AR_ - *HSP70A-RBCS2* promoter; cCA, ARS2, GLE, sec - secretion signals; SP20 - serine/proline glycomodule; T_RPL23_ - *RPL23* terminator; PF/Fu-S - prefusion stabilized spike protein lacking the furin cleavage site. **(C)** Immunodetection of HA-tagged spike proteins in transformants generated with the constructs shown in **(B)**. CL - cell lysates; CM - TCA-precipitated proteins from 1.7 mL of culture medium; WT - wild type; *cross-reaction. **(D)** Quantification of Chlamydomonas-produced spike protein. Three independent preparations of PF/Fu-S protein precipitated with TCA from 1.7 mL culture medium (CM) and increasing amounts (5 to 25 ng) of purified recombinant mCherry carrying a C-terminal HA tag were analyzed by immunoblotting. **(E)** Secretion kinetics of PF/Fu-S. Cultivation was started by diluting a stationary phase PF/Fu-S culture and cells and culture medium were harvested at the indicated time points during cultivation for immunoblot analyses. Cell counts and signal ratios of PF/Fu-S (HA antibody) to plastidic CPN60A are plotted over time (*n* = 3, error bars indicate SD). **(F)** Exemplary immunoblot of PF/Fu-S and CPN60A in culture medium (CM, 1.7 mL) and cell lysates (CL, 2 μg chlorophyll) used for calculating the HA/CPN60A ratios shown in **(E)**.

The 1,212-amino acids N-terminal portion of the SARS-CoV-2 spike protein lacking the membrane anchor (UniProt: P0DTC2) was reverse translated using the most commonly used Chlamydomonas codons. For stable foreign gene expression, introns need to be inserted into the coding region with defined flanking sequences, generating exons of at most 500 bp (Baier et al., 2018b). To meet these requirements, the first *Chlamydomonas RBCS2* intron was inserted six times with the flanking sites GAG/intron/G, the second intron was inserted twice with flanking sites GCG/intron/GC, and the third intron was inserted once using ACG/intron/G as flanking sites. A single internal *Bsa*I recognition site was removed by changing the used codon for threonine (ACC) into ACG. The sequence was split into two fragments, which were flanked with *Bsa*I recognition sites such that, upon *Bsa*I digestion, fragments with AATG and AGGT or AGGT and TTCG overhangs are generated for the B3 and B4 positions of level 0 parts according to the MoClo syntax for plant genes (Weber et al., 2011; Crozet et al., 2018) (Figure 1A). Synthesis and cloning into the pUC57 vector was done by BioCat (Heidelberg), resulting in level 0 parts pMBS704 (*secCoV2-S up*) and pMBS705 (*CoV2-S down*). To remove the sequence coding for the N-terminal endogenous secretion signal, pMBS704 was used as template for PCR using primers 5′-AAAGAAGACAAAATGAACCTGACCACCCGCACCCAGC-3′ and 5′-AAAGAAGACTTCATTTGAGACCTTTATATCTAG ATG-3′. The resulting 5,170-bp PCR product was digested with *Bbs*I and assembled by ligation (the MoClo reaction conditions were: 5 h at 37°C, 5 min at 50°C and 10 min at 80°C), giving rise to level 0 part pMBS706 (*CoV2-S up*). pMBS705 was used as template for site directed mutagenesis PCR to replace codons for the furin cleavage site (S1/S2) “RRAR” with codons for “GSAS,” as described previously (Wrapp et al., 2020; Papa et al., 2021) and to replace the codons for “KV” with two proline codons, as described previously (Pallesen et al., 2017; Wrapp et al., 2020). The following primer combinations were chosen to amplify three fragments (overhangs are underlined, lower case letters indicate base changes): 5′-AAAGAAGACGTCTCAAGGTGCCCGTGGCCA-3′ and 5′-AAAGAAGACTTtaCcGGGGCTGTTGGTCTGGGTCTGG-3′ (218 bp), 5′-AAAGAAGACAAgGtaGCGCtaGCAGCGTGGCC AGCCAGAGCA-3′ and 5′-AAAGAAGACTTGTCGTTCAG CACGCTGCTGATGGC-3′ (1,390 bp), and 5′-AAAGAAGA CAACGACATCCTGAGCCGCCTGGACcccccGGAGGTGAG CTTGC-3′ and 5′-AAAGAAGACTTCTCGCGAACCCCACTT GATGTACT-3′ (1,302 bp). The three PCR products were combined with pAGM9121 (Weber et al., 2011), digested with *Bbs*I and directionally assembled by ligation into the level 0 part pMBS708 (*CoV2-SGSAS/PP-down*). A level 0 part encoding the arylsulfatase 2 secretion peptide (*ARS2*) was generated via the annealing of oligonucleotides 5′-GAAGACAACCATGAGCCTGGCCACCCGCCGCTTCGGCG CCGCCGCCGCCCTGCTGGTGGCCGCCTGCGTGCTGTGC ACCGCCCCCGCCTGGGCAATGAAGTCTTC-3′ and 5′-GAAGACTTCATTGCCCAGGCGGGGGCGGTGCACAGCA CGCAGGCGGCCACCAGCAGGGCGGCGGCGGCGCCGAA GCGGCGGGTGGCCAGGCTCATGGTTGTCTTC-3′. The product was combined with destination vector pAGM1276 (Weber et al., 2011), digested with *Bbs*I and ligated to yield pMBS489 (*spARS2*). The sequence encoding the secretion signal of the gamete lytic enzyme (*GLE*) was similarly produced via the annealing of oligonucleotides 5′-GAAGACAACCATGAGCCTGGCCACCCGCCGCTTCGGCG CCGCCGCCGCCCTGCTGGTGGCCGCCTGCGTGCTGTGC ACCGCCCCCGCCTGGGCAATGAAGTCTTC-3′ and 5′-GAAGACTTCATTGCCCAGGCGGGGGCGGTGCACAGCAC GCAGGCGGCCACCAGCAGGGCGGCGGCGGCGCCGAAG CGGCGGGTGGCCAGGCTCATGGTTGTCTTC-3′ followed by digestion with *Bbs*I and ligation with destination vector pAGM1276 (Weber et al., 2011), giving rise to level 0 part pMBS490 (*spGLE*). A level 0 part encoding a hemagglutinin (HA) motif, an RGS linker and a octa-histidine tag was obtained via the annealing of oligonucleotides 5′-AAGAAGACAATTCGTCTGGTTACCC C T A CGACGTGCCC GACTACGCTCGCG G C A GCCACCACCACCACCACCACCA CCACTAAGCTTAAGTCTT C A A-3′ and 5′- T T G A A G A C T T AAGCTTAGTGGTGGTGGTGGTGGTGGTGGTGGCTGCCG C G A G C G T A G T C G G G C A C G T C G T A G G G G T A A C C AGA CGAATTGTCTTCTT-3′. Digestion of the annealing product and the destination vector pAGM1301 with *Bbs*I and ligation yielded pMBS723 (*1xHA-8xHis*). The SP20 glycomodule (Ramos-Martinez et al., 2017) coupled to a triple HA motif was reverse translated using the most commonly used Chlamydomonas codons and flanked with *Bsa*I recognition sites, producing overhangs for the B5 position according to the MoClo syntax for plants (Weber et al., 2011; Crozet et al., 2018). Synthesis and cloning into the pUC57 vector were done by BioCat (Heidelberg), yielding level 0 part pMBS514 (*SP20-3xHA*). Plasmid pMBS514 was used as template to replace the sequence encoding two HA motifs by a sequence encoding the RGS-8xHis motif by PCR using primers 5′-TAAGCTTTGAGACCTTCCAATATC-3′ and 5′- TCATTAGTGGTGGTGGTGGTGGTGGTGGTGGCTACCGC GAGCGTAGTCGGGCACGTCGTA-3′. The 2,810-bp PCR product was phosphorylated with polynucleotide kinase (NEB) and circularized with T4 DNA ligase (NEB) as described previously (Gupta et al., 2021), generating pMBS659 (*SP20-1xHA-8His*). All PCRs were conducted with Q5 High-Fidelity Polymerase (NEB) following the manufacturer’s instructions. Sequences of the level 0 parts were verified by Sanger sequencing (Seqlab). All parts are available from the Chlamydomonas Resource Center. Level 1 module assembly was performed by combining newly constructed level 0 parts with level 0 parts (pCM) from the Chlamydomonas MoClo toolkit (Crozet et al., 2018) and destination vector pICH47742 (Weber et al., 2011). For directional assembly, the following level 0 parts were chosen: A1-B1-pCM0-015 (*HSP70A-RBCS2* promoter + 5′-untranslated region (UTR)); A1-B2-pCM0-017 (*HSP70A-RBCS2* promoter + 5′-UTR); B2-pCM0-051 (*spcCA*); B2-pMBS489 (*spARS2*); B2-pMBS490 (*spGLE*); B3-pMBS704 (*CoV2-S up*); B2-B3-pMBS706 (*secCoV2-S up*); B4-pMBS705 (*CoV2-S down*); B4-pMBS708 (*CoV2-SGSAS/PP-down*); B5-pMBS723 (*1xHA-8xHis*); B5-pMBS514 (*SP20-3xHA*); B5-pMBS659 (*SP20-1xHA-8xHis*); B6-C1-pCM0-119 (*RPL23 3′*-UTR). The individual level 0 parts were released by *Bsa*I digestion and assembled into five level 1 modules using T4 DNA ligase according to Figure 1B. The level 1 modules were then combined with pCM1-01 (level 1 module with the *aadA* gene conferring resistance to spectinomycin flanked by *PSAD* promoter and terminator) from the Chlamydomonas MoClo kit (Crozet et al., 2018), with plasmid pICH41744 containing the proper end-linker, and with destination vector pAGM4673 (Weber et al., 2011), digested with *Bbs*I, and ligated to yield the five level 2 devices displayed in Figure 1B.

### Cloning and purification of recombinant mCherry-6His-1xHA

The mCherry coding sequence was amplified via PCR using plasmid CD3-960 (Nelson et al., 2007) as template and primers 5′-AAAAGCTAGCAAAGAATTCATGGTGAGCAAGGGCGAGG AG-3′ and 5′-TTTCTCGAGTTTGTACAGCTCGTCCATGC-3′ flanked by *Nhe*I and *Xho*I recognition sites. The PCR product and pET21b were digested with *Nhe*I and *Xho*I, gel-purified, and ligated to yield pET-mCherry-6xHis (pMS1034). pMS1034 was used as a template for PCR to add the coding sequence for the 1xHA motif via primers 5′-AAAAGGATCCTATCCGTATGATGTGCCAGATTATGCCT GAGATCCGGCTGCTAACAAA-3′ and 5′-TTTTGGATCC GTGGTGGTGGTGGTGGTGCTCGAG-3′ (*Bam*HI sites are underlined). The resulting 6,140 bp PCR product was digested with *Bam*HI and recircularized, giving rise to pET21b-mCherry-6xHis-1xHA (pMS1090). mCherry-6xHis-1xHA was expressed in ER2566 cells in LB-medium (supplemented with 100 μg/mL ampicillin (K029.2, Carl Roth)) after inducing the expression with 0.5 mM IPTG at 37°C for 4 h and purified under denaturing conditions as described previously (Hammel et al., 2018) using nickel-charged resin. The concentration of recombinant mCherry-6xHis-1xHA was determined using the Pierce BCA protein assay kit following the manufacturer’s instructions.

### Protein analyses by SDS-PAGE

For whole-cell protein extraction, cells were pelleted and resuspended in 50 mM DTT, 50 mM Na_2_CO_3_, 2.5% (w/v) SDS, and 15% (w/v) sucrose (pH 9.3), boiled for 2 min at 95°C and centrifuged. After determination of chlorophyll content (Porra et al., 1989), a sample volume corresponding to 2 μg chlorophyll (roughly 15 μg protein) was subjected to SDS-PAGE. For analyzing proteins secreted into the culture medium, cells were grown to stationary phase. Next, cells in 1.7 mL culture were pelleted and the supernatant was transferred to a 2-mL reaction tube. Trichloroacetic acid (TCA) was added to a final concentration of 10%. The samples were incubated for 30 min on ice and centrifuged at 15,000 g for 15 min at 4°C. The pellet was washed with 500 μL ice-cold acetone, resuspended in 20 μL of 75 mM Tris-HCL pH 6.8, 2.5% (w/v) SDS, 50 mM DTT and 10% (v/v) glycerol, boiled for 2-5 min at 95°C and subjected to SDS-PAGE on 8% polyacrylamide gels. After semidry blotting, immunodetection was performed by enhanced chemiluminescence using an INTAS imaging system. Primary antibodies used for immunodetection were mouse anti-HA (H9658, Sigma-Aldrich, 1:10,000), mouse anti-RBD (MAB105401, R&D Systems, 1:2,000) and mouse anti-ACE2 (sc-390851, Santa Cruz Biotechnology, 1:2,000). The secondary antibody was m-IgGκ BP-HRP (sc-516102, Santa Cruz Biotechnology, 1:10,000). Densitometric band quantifications after immunodetections were done with the FUSIONCapt Advance program (PEQLAB) using two lanes for the PF/Fu-S and mCherry-6xHis-1xHA and a linear background subtraction. The molecular weight was also determined with the FUSIONCapt Advance program (PEQLAB) using the standard settings adapted to the protein marker used.

### Nickel-nitrilotriacetic acid purification of SARS CoV-2 spike protein from culture medium

A total of 250 mL of culture medium of a PF/Fu-S transformant grown to stationary phase were loaded onto an affinity column containing 500 μL nickel-charged resin equilibrated with PBS (137 mM NaCl, 2.7 mM KCl, 81 mM Na_2_HPO_4_, 18 mM KH_2_PO_4_, pH 7.6). After two washing steps with 5 mL PBS containing 5 mM imidazole, bound proteins were eluted with 1.5 mL PBS containing 500 mM imidazole and three fractions of 0.5 mL each were collected.

### Ammonium sulfate precipitation of secreted proteins

Cells from 200 mL of culture of a PF/Fu-S transformant grown to stationary phase were removed by three subsequent centrifugations at 4,000 g for 5 min at 25°C. The supernatant was transferred to a 500-mL beaker and ammonium sulfate was slowly added under continuous stirring until the solution was saturated (106.6 g/200 mL). Stirring was continued for another 30 min. Precipitated proteins were pelleted by centrifugation at 4000 g for 45 min at 4°C and the supernatant was carefully removed. The pellet was resuspended in 5 mL PBS and dialyzed overnight against PBS (ZelluTrans 3.5 MWCO, Carl Roth). Finally, 1x protease inhibitor cocktail (cOmplete*™*, EDTA-free Protease Inhibitor Cocktail, Roche) was added to the solution according to the manufacturer’s instructions.

### SARS-CoV-2 spike protein binding assay to recombinant hACE2

Recombinant hACE2 with a C-terminal deca-histidine tag (SAE0064, Sigma-Aldrich) was resuspended in PBS to a final concentration of 50 ng/μL. 1 μg of hACE2 was mixed with 300 μL of proteins recovered after ammonium sulfate precipitation from a PF/Fu-S culture and incubated for 30 min at 37°C. The protein mixture was filled up to 1 mL with PBS, and 66 μL of nickel affinity resin (equilibrated with PBS) and 1x protease inhibitor cocktail were added. After an incubation for 30 min on a circular rotator, samples were cooled for 10 min on ice, centrifuged at 100 g for 30 s at 4°C and washed with 1 mL PBS containing 5 mM imidazole. Elution of bound proteins was performed with 50 μL PBS containing 500 mM imidazole. Flow-through, washing and elution fractions were TCA precipitated as described above.

### SARS-CoV-2 spike protein binding assay to cell-based hACE2

293T + AT and 293T cells were seeded into 6-well plates and grown until they reached 70% confluence. The medium was partially removed and 500 μL of proteins, recovered after ammonium sulfate precipitation from a PF/Fu-S culture, were applied. After 1 h incubation at 37°C and 5% CO_2_, the medium was removed and cells gently resuspended in PBS. After centrifugation at 800 g for 10 min at 4°C, the supernatant was removed and the cell pellet lysed in ice-cold RIPA buffer (150 mM NaCl, 50 mM Tris pH 7.4, 1% Nonidet P-40, 0.1% SDS, 0.5% sodium deoxycholate, 5 mM EDTA) containing 1x protease inhibitor cocktail. Cell debris was removed by centrifugation (8,000 g, 10 min, 4°C). For immunoblot analysis, the lysates were separated on an SDS-polyacrylamide gel and transferred to a nitrocellulose membrane. Membranes were blocked and incubated with specific antibodies: against HA-tag, RBD, and ACE2 with the specifications listed above.

## Results

### Production and secretion of the full-length ectodomain of the SARS-CoV-2 spike protein in *Chlamydomonas reinhardtii*

To engineer Chlamydomonas toward the production and secretion of the SARS-CoV-2 spike protein, we reverse translated its amino acid sequence with optimal Chlamydomonas codon usage. We included the endogenous secretion peptide but excluded the C-terminal membrane anchor (Zhu et al., 2020). To improve nuclear transgene expression, we subsequently inserted the three introns of the Chlamydomonas *RBCS2* gene into the coding region, as proposed previously (Baier et al., 2018b; Schroda, 2019). To facilitate gene synthesis, the sequence was divided into a 2610-bp and a 2851-bp fragment. Both fragments were flanked by *Bsa*I recognition sites, following the syntax of the Chlamydomonas MoClo toolkit (Crozet et al., 2018) for standardized level 0 parts in position B3 (*sec CoV2-S up)* and B4 (*CoV2-S down*) (Figure 1A). We first wanted to test whether Chlamydomonas can produce and secrete the spike protein via its endogenous secretion signal. To this end, we assembled the two spike protein gene fragments with the strong *HSP70A-RBCS2* promoter, the coding region for C-terminal HA- and octa-histidine tags, and the *RPL23* terminator. The resulting level 1 module was then assembled with the spectinomycin resistance cassette *aadA* into a level 2 multigene device (Figure 1B, WT-S). Immunoblot analyses of cell lysates from a spectinomycin resistant transformant generated with WT-S revealed a protein migrating above the 130 kDa marker that was specifically detected by the HA antibody (Figure 1C) and roughly matched the expected mass of 137 kDa. However, no signal was obtained for proteins from the culture medium (not shown), indicating that the endogenous secretion peptide is not functional in Chlamydomonas.

To enable secretion of the spike protein, we added the coding regions for three different N-terminal secretion signals of native extracellular Chlamydomonas proteins, namely carbonic anhydrase (cCA) (Lauersen et al., 2013), arylsulfatase 2 (ARS2) (Eichler-Stahlberg et al., 2009) and gamete lytic enzyme (GLE) (Ramos-Martinez et al., 2017; Figure 1B). Additionally, the coding region for a C-terminal synthetic glycomodule of 20 serine/proline repeats was added, which was reported to enhance secretion yields in Chlamydomonas (Ramos-Martinez et al., 2017). To increase detectability, the glycomodule was equipped with a triple HA motif (SP20 3xHA). Immunoblot analysis of proteins precipitated from the culture medium of spectinomycin resistant transformants generated with the revised constructs revealed specific protein bands of ∼229, ∼117 and ∼90 kDa (Figure 1C). Signals were strongest for spike proteins with secretion signals from carbonic anhydrase and arylsulfatase. The mature native spike protein expressed in human cells has a calculated molecular mass of 139.5 kDa, but an apparent molecular mass of ∼200 kDa on SDS gels (Walls et al., 2020; Wrapp et al., 2020), with the difference of ∼60 kDa likely caused by the 22 N-linked glycans (Watanabe et al., 2020). The calculated mass of the mature Chlamydomonas-produced spike protein is 142 kDa. Since O-glycosylation at the SP20 glycomodule has been shown to result in an increase of the apparent molecular mass of ∼23 kDa in Chlamydomonas (Ramos-Martinez et al., 2017), it appears likely that the protein migrating at ∼229 kDa represents the N- and O-glycosylated full-length spike protein ectodomain. The latter appears to be efficiently processed, giving rise to two C-terminal fragments, accounting for the weak signal for the full-length ectodomain migrating at ∼229 kDa and the strong signals at ∼117 and ∼90 kDa. In the weakly expressing GLE-S transformants, the full-length ectodomain appears to be below the detection limit and only the two C-terminal cleavage products are detected (Figure 1C).

During its synthesis in human cells, the SARS-CoV-2 spike protein is cleaved into two functional subunits S1 and S2 by a furin-like protease in the Golgi compartment (Cai et al., 2020; Hoffmann et al., 2020a; Figure 1A). Cleavage at the S1/S2 boundary is strongly enhanced by the presence of a “RRAR” furin cleavage site that is missing in the SARS-CoV spike protein (Hoffmann et al., 2020a,b; Walls et al., 2020; Wrapp et al., 2020; Papa et al., 2021). To test, whether processing of the spike protein produced in Chlamydomonas was also mediated by a furin-like protease, we first replaced sequences coding for the N-terminal endogenous signal peptide by that of cCA (Figures 1A,B). Next, we replaced sequences encoding the furin cleavage site R_682_RAR_685_ by sequences coding for G_682_SAS_685_, which inactivates the cleavage site (Wrapp et al., 2020; Papa et al., 2021). We also substituted codons for K_986_V_987_ by codons for proline, which stabilizes the prefusion conformation of spike proteins, thus increasing expression yields and immunogenicity (Pallesen et al., 2017; Wrapp et al., 2020). Finally, we added sequences encoding the SP20 glycomodule, a single HA tag, and an octa-histidine tag (PF/Fu-S in Figure 1B). Immunoblot analysis of proteins precipitated from the culture medium of a spectinomycin-resistant PF/Fu-S transformant revealed a major protein band at ∼233 kDa and minor ones at ∼117 kDa and ∼90 kDa (Figure 1C). Among the modifications made, it is most likely the elimination of the furin cleavage site that almost completely abolished proteolytic cleavage of the spike protein during its passage through the secretory pathway, as has been observed in human cells (Hoffmann et al., 2020b; Walls et al., 2020; Papa et al., 2021). To estimate the concentration of the secreted spike protein ectodomain, we performed quantitative immunoblotting. For this, proteins in the culture medium of a PF/Fu-S transformant were precipitated and analyzed next to known amounts of recombinant HA-tagged mCherry by immunoblotting using an anti-HA antibody (Figure 1D). We estimated a concentration of 11.2 ± 1.8 μg/L (*n* = 3, ± SD) for the spike protein.

We next wanted to assess when during cultivation the spike protein is secreted and whether it accumulates in cells prior to secretion. Moreover, we wanted to verify that spike protein in the medium is derived from secretion and not from cell lysis. For these ends, we diluted a stationary phase culture of a PF/Fu-S transformant and harvested cells and culture medium at different time points during cultivation until the culture re-entered stationary phase. Spike protein and CPN60A in cells and culture medium were detected by immunoblotting (CPN60A was chosen because it is an abundant, soluble, plastidic protein). As shown in Figures 1E,F, we found that the spike protein only accumulated in cells coming from or entering stationary phase. Spike protein in the culture medium was detected virtually only when cells had re-entered stationary phase. CPN60A, apparently derived from cell lysis, appeared in the medium already in late log phase. There, its abundance declined when that of the spike protein was highest, indicating that the spike protein is derived from secretion and not from cell lysis.

### SARS-CoV-2 spike protein from Chlamydomonas cannot be purified by IMAC but is readily concentrated by ammonium sulfate precipitation

**FIGURE 2:**
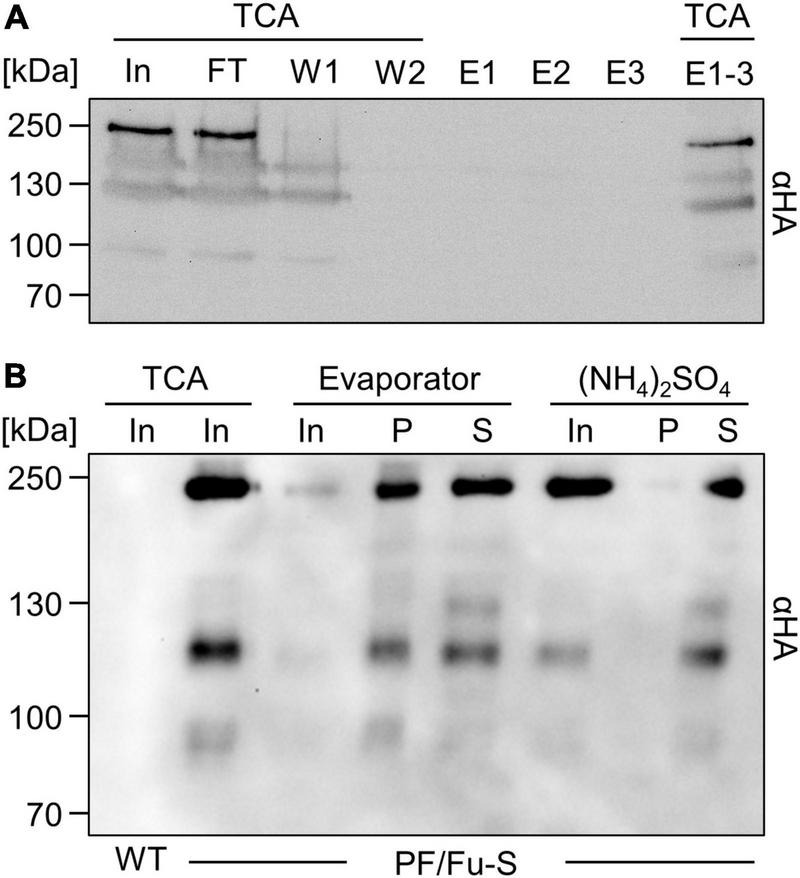
Enrichment of secreted SARS-CoV-2 spike protein from Chlamydomonas culture medium. **(A)** Attempted purification of octa-histidine-tagged PF/Fu-S protein from 250 mL culture medium via nickel affinity chromatography. Proteins in 1.7 mL of input (In), flow-through (FT), and washes (W1 and W2), and those in the entire eluate (E1-3) were precipitated with TCA. TCA precipitates and 20 μL of three elution fractions (E) were analyzed by immunoblotting. **(B)** Spike protein enrichment. Proteins in 100 mL of culture medium of wild-type (WT) and a PF/Fu-S producing transformant were precipitated with TCA (In, 85x enriched), concentrated by a rotary evaporator (In, 17x enriched) and centrifugal filters (34x enriched), or precipitated with (NH_4_)_2_SO_4_ (In, 34x enriched). Enriched proteins were centrifuged to separate aggregated (P) and soluble (S) proteins. All proteins were analyzed by immunoblotting.

After the successful production of the full-length spike protein ectodomain in Chlamydomonas, we aimed to purify it from culture medium via its C-terminal octa-histidine tag. However, the spike protein failed to bind to the nickel resin and largely remained in the flow through (Figure 2A). The same result was obtained when the medium was supplied with 2% Triton or 250 mM NaCl, with longer incubation times, or when we used cobalt beads instead of nickel beads. An affinity purification with immobilized HA antibodies also failed (not shown). To enrich the protein by other means, we concentrated proteins in the culture medium with a rotary evaporator, followed by two runs through centrifugal filters. Although an enrichment of the spike protein was achieved, about half of it was found in aggregates (Figure 2B). As an alternative, we precipitated proteins in the culture medium with ammonium sulfate and could recover virtually all of the spike protein, with very little found in aggregates (Figure 2B). Hence, ammonium sulfate precipitation appears most suitable for enrichment.

### SARS-CoV-2 spike protein from Chlamydomonas binds to the hACE2 receptor

**FIGURE 3:**
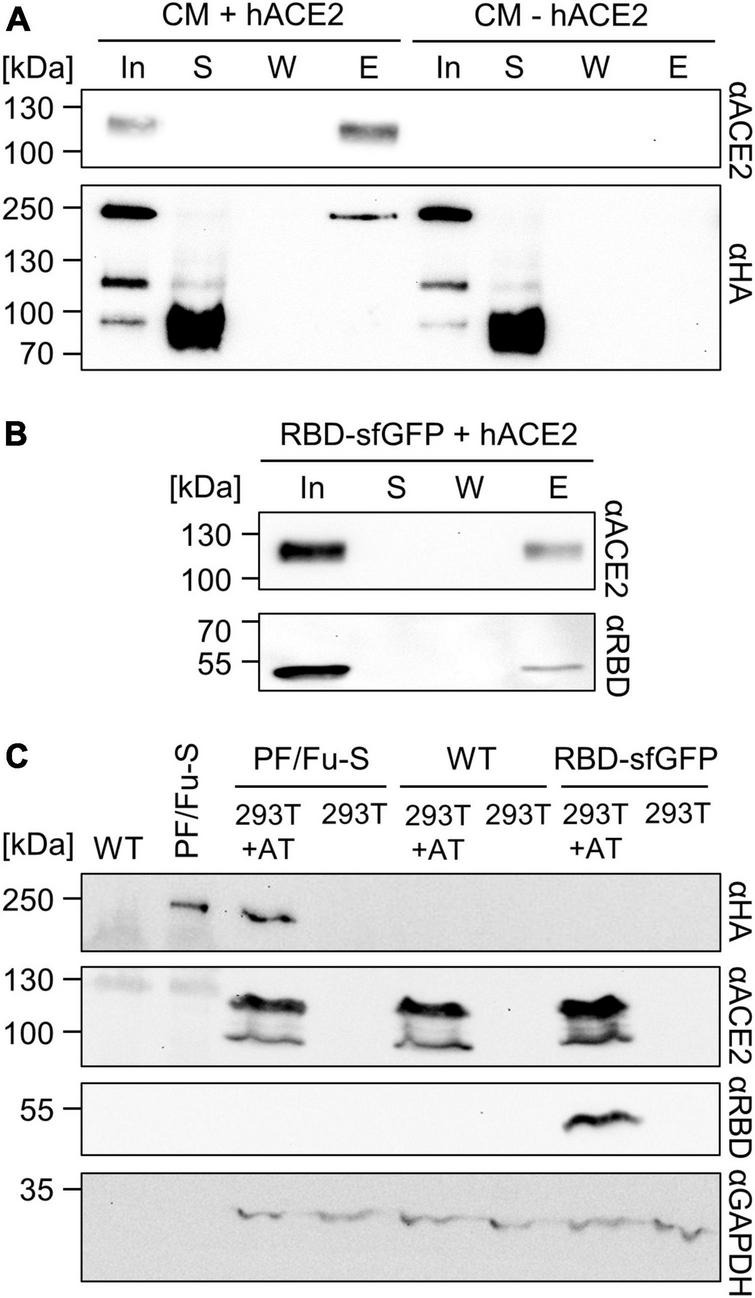
Assays for testing the functionality of SARS-CoV-2 spike protein produced in Chlamydomonas. **(A)** *In vitro* pull-down assay. PF/Fu-S enriched from culture medium (CM) was incubated with (+) or without (–) hACE2-10xHis and incubated with nickel-NTA resin. S - supernatant; W - wash; E - eluate (all proteins in each fraction loaded after TCA precipitation). In - 10% of input. **(B)** Assay as in A) but using human-derived RBD-sfGFP. **(C)** Cell-based functionality assay. Enriched PF/Fu-S and RBD-sfGFP were applied to 293T cells overexpressing hACE2 and hTMPRSS2 (293T + AT) and regular 293T cells. Cells were washed, lysed, and total proteins analyzed by immunoblotting. As control, proteins enriched from WT culture medium were employed. GAPDH serves as endogenous control. All proteins were analyzed by immunoblotting.

Next, we wanted to test the functionality of the spike protein produced in Chlamydomonas. The ability of the protein to bind to the human ACE2 receptor (hACE2) was described previously (Hoffmann et al., 2020b; Letko et al., 2020) and binding to ACE2 is an accepted assay to verify the functionality of recombinant spike protein (Chuck et al., 2009). We incubated proteins enriched by ammonium sulfate precipitation from a PF/Fu-S culture with or without recombinant hACE2 containing a C-terminal deca-histidine tag. The hACE2 used is soluble as it only contains the topological part of the protein (Q_18_-S_740_) lacking a transmembrane domain. Nickel resin was added and bound proteins eluted. As shown in Figure 3A, Chlamydomonas-produced spike protein co-eluted with hACE2. No spike protein was eluted if hACE2 was absent. Notice that less spike protein was recovered with hACE2 than supplied in the input, indicating that only part of it bound to hACE2. Unbound spike protein in the supernatant was largely converted to the ∼90-kDa breakdown product during the 30-min incubation at 37°C, possibly due to proteases in the supernatant that were particularly active at the elevated temperatures (Figure 3A). As a control, we produced the SARS-CoV-2 RBD fused to sfGFP in transiently transfected human 293T cells. Secreted proteins were concentrated with centrifugal filters and incubated with recombinant hACE2. Like Chlamydomonas-produced spike protein, RBD-sfGFP co-eluted with hACE2 (Figure 3B).

To complement the *in vitro* binding assay, we performed a second functionality assay based on the binding of spike protein to hACE2 on human cells. For this, we concentrated secreted proteins from culture medium of a PF/Fu-S transformant by ammonium sulfate precipitation. Concentrated proteins were then applied to 293T cells constitutively overexpressing hACE2 and hTMPRSS2 (293T + AT) and, as a control, to regular 293T cells, both in the presence of 10% fetal calf serum. In contrast to the *in vitro* assay, hACE2 used here is the membrane-bound protein on the cell surface. Importantly, we did not observe any cytotoxic or other negative effects of this treatment on the cells by microscopy. After washing, cells were lysed and proteins in the lysates analyzed by immunoblotting. Spike protein was detected in lysates from cells expressing hACE2, while it was not detected in lysates from cells lacking hACE2 (Figure 3C). The same result was obtained with RBD-sfGFP. No signal for the spike protein was obtained if cells were treated with concentrated secreted proteins from wild-type cells. Taken together, these results provide strong evidence that the full-length ectodomain of the SARS-CoV-2 spike protein produced in Chlamydomonas is functional.

## Discussion

Here we report on the construction of Chlamydomonas strains that secrete the functional full-length SARS-CoV-2 spike protein ectodomain into the medium. To our knowledge, this has only been achieved with mammalian and insect cell-based expression systems. Recent reports on the expression of SARS-CoV-2 spike protein in Chlamydomonas and the diatom *P. tricornutum* were limited to intracellularly expressed RBD (Berndt et al., 2021; Malla et al., 2021; Slattery et al., 2022). The advantage of Chlamydomonas is that it is a GRAS organism that can be cultivated at low cost in simple media at large scale, making Chlamydomonas an attractive production platform for human therapeutical proteins in low-income countries (Rosales-Mendoza et al., 2020).

During our endeavor to produce the spike protein in Chlamydomonas, we have learned that its native signal peptide appears not to guide the protein to the secretory pathway in Chlamydomonas. For this, a signal peptide of a native Chlamydomonas protein was required, with those from cCA and ARS2 being more efficient than that of GLE (Figure 1C). We observed that during its passage through the secretory pathway the spike protein appears to be processed at the multi-basic furin cleavage site, similar to mammalian cells (Hoffmann et al., 2020b; Walls et al., 2020; Papa et al., 2021), which might point to the presence of a furin-like protease in the Golgi compartment in Chlamydomonas. An O-glycosylation was reported at T_678_ close to the furin cleavage site with a potential role in regulating cleavage (Sanda et al., 2021). If so, the efficient cleavage observed in Chlamydomonas might indicate that this O-glycosylation is realized in Chlamydomonas.

A C-terminal glycomodule of 20 serine-proline repeats (SP20) apparently was required for efficient spike protein secretion, as we failed to identify transformants secreting the protein if it contained Chlamydomonas secretion signals but not the SP20 module (not shown), corroborating earlier findings on the stimulating effect of the SP20 module on protein secretion in Chlamydomonas (Ramos-Martinez et al., 2017). Purification of the spike protein via an HA or an octa-histidine tag at the very C-terminus failed, albeit we could detect the HA tag in immunoblots (Figure 2A). A possible reason for the unsuccessful affinity purification could be steric hindrance of binding by an unfavorable folding of the tag or by glycans at the SP20 module. Alternatively, the tag might be proteolytically truncated or somehow modified during its passage through the secretory pathway, as was observed for RBD produced in the diatom *P. tricornutum* (Slattery et al., 2022).

Concentration of secreted proteins by a rotatory evaporator and centrifugal filters led to aggregation of part of the spike protein, while enrichment by ammonium sulfate precipitation resulted in soluble spike protein (Figure 2B). Functionality of the Chlamydomonas-produced spike protein was deduced from its ability to interact with soluble recombinant ACE2 and ACE2 expressed on human cells (Figure 3). Since glycosylation has been shown to be essential for the correct folding of soluble and functional spike protein (Rossen et al., 1998; Chuck et al., 2009) and the apparent mass shift by N-glycosylation of ∼60 kDa observed for spike protein produced in human cells (Walls et al., 2020) was roughly observed also for the Chlamydomonas-produced protein, it appears that N-glycosylation of the secreted spike protein is realized in Chlamydomonas and promotes proper spike protein folding. Nevertheless, glycoproteomic analyses on the Chlamydomonas-produced spike protein will have to be performed in future work to prove successful N-glycosylation. The production of functional secreted spike protein in Chlamydomonas is in line with recent reports on the production of functional forms of human vascular endothelial growth factor (Chavez et al., 2016), human epithelia growth factor (Baier et al., 2018a), and RBD (Berndt et al., 2021) in this alga, indicating that Chlamydomonas is a suitable production platform for human therapeutic proteins. However, it is important to keep in mind that glycans on secreted proteins differ between Chlamydomonas and humans. For example, Chlamydomonas (and plant) N-glycans contain core β1,2-xylose and α1,3-fucose that are absent in humans (Mathieu-Rivet et al., 2020). However, glycans on plant-derived vaccines did not provoke adverse events after vaccination in humans and rhesus macaques and the allergenic nature of some plant glycoproteins was proposed to be driven by the protein backbone rather than the glycan motifs (Ward et al., 2014, 2021; Pillet et al., 2022). Uncommon for plants, N-glycans in Chlamydomonas carry a second, terminal xylose and modifications of mannose residues with one 6-O-methylation (Mathieu-Rivet et al., 2020). Potential immunogenic effects of such O-methylated N-glycans need to be investigated and may require the construction of strains lacking the responsible O-methyltransferases (Mócsai et al., 2019; Mathieu-Rivet et al., 2020). Potential immunogenic effects of the O-glycosylated SP20 module also need to be investigated and it may be necessary to proteolytically remove the SP20 module after enrichment of the secreted glycoprotein.

The assembly and testing of the constructs for expressing the various spike proteins could be realized rapidly in iterative cycles thanks to the MoClo strategy, easy transformation and short generation times of Chlamydomonas, and the use of expression strains (Weber et al., 2011; Crozet et al., 2018; Schroda, 2019; Neupert et al., 2020; Schroda and Remacle, 2022). Clearly, these features will facilitate adaptations of the spike protein to SARS-CoV-2 mutant variants and help addressing the remaining bottlenecks associated with spike protein production in Chlamydomonas: first, we need to find a method for purifying the protein from the culture medium, *e.g.*, by changing the position of tags to the N-terminus, or by employing other tags like GST, MBP, FLAG, or StrepII. Second, we need to enhance yields. By quantitative immunoblotting we estimated a yield for secreted spike protein of 11.2 μg/L (Figure 1D). Because the large, glycosylated spike protein will be less efficiently transferred to nitrocellulose by semi-dry blotting than the small non-glycosylated mCherry used as standard, we are definitely underestimating yields. LC-MS/MS-based methods based, *e.g.*, on QconCATs (Hammel et al., 2018) should be employed in future. Reported yields of secreted proteins produced in Chlamydomonas were 100 μg/L for human erythropoietin (Eichler-Stahlberg et al., 2009), 28 μg/L for human vascular endothelial growth factor (Chavez et al., 2016), 100 μg/L for a human epithelial growth factor fusion with luciferase (Baier et al., 2018a), 0.7 mg/L for various reporter proteins (Lauersen et al., 2015b), 15 mg/L for mVenus (Ramos-Martinez et al., 2017), and 10-12 mg/L for luciferase alone or fused to an ice-binding protein (Lauersen et al., 2013, 2015a). Hence, our yields are in the same range as those reported for other human therapeutic proteins but far below those reported for the simpler reporter proteins. We observed that apparently fully glycosylated spike protein accumulated in cells and culture medium only when cells had entered the stationary phase, while it was not detectable in log-phase cells (Figures 1E,F). Possibly, the massive production of glycoproteins in growing and dividing log-phase cells competes with the production of the large spike protein with its 22 N-linked glycans and its SP20 glycomodule. Not only the N- and O-glycosylation capacity might be limiting here, but also the chaperoning capacity for proteins that are difficult to fold. Accordingly, the ER chaperone BiP was found as a major contaminant of spike protein preparations (Cai et al., 2020). Therefore, enhanced production of the spike protein (and other human proteins) in Chlamydomonas might be achieved by the overexpression of components of the ER protein folding machinery, or of the bZIP1 transcription factor regulating the Chlamydomonas ER unfolded protein response (Gasser et al., 2008; Yamaoka et al., 2019). Moreover, protein secretion yields in Chlamydomonas can be strongly improved by optimizing culture conditions (Lauersen et al., 2015a; Freudenberg et al., 2021).

In conclusion, the successful production and secretion of the functional full-length ectodomain of the spike protein in Chlamydomonas underpins the potential of this alga as a new platform for the production of therapeutic glycoproteins in low-income countries. However, unresolved issues related to low yield, unsuccessful affinity purification, and potential immunogenic effects of O-methylated N-glycans remain to be addressed in future research.

## Data availability statement

The datasets presented in this study can be found in online repositories. The names of the repository/repositories and accession number(s) can be found in the article/supplementary material.

## Author contributions

AK, JN, and AP generated all constructs and performed all experiments. AK, JN, GE, and MS conceived and supervised the project. MS, AK, and JN wrote the article with contributions from all authors. All authors contributed to the article and approved the submitted version.

## Abbreviations

MoClo: Modular Cloning
RBD: receptor binding domain
ACE: angiotensin-converting enzyme
GRAS: generally recognized as safe
HEK: human embryonic kidney
sfGFP: superfolder GFP
HA: hemagglutinin.
